# Comparison of the efficacy and safety of TAF, TDF, and LdT to prevent the transmission of hepatitis B in pregnant women: A retrospective study

**DOI:** 10.1002/iid3.1204

**Published:** 2024-02-27

**Authors:** Shufang Pan, Ying Zhang, Yingfu Zeng, Chaoshuang Lin

**Affiliations:** ^1^ Department of Infectious Diseases The Third Affiliated Hospital of Sun Yat‐Sen University Guangzhou Guangdong Province China

**Keywords:** hepatitis B virus, immune tolerance, preventing mother‐to‐child transmission, telbivudine, tenofovir alafenamide fumarate, tenofovir disoproxil fumarate

## Abstract

**Objective:**

To compare the efficacy and safety of telbivudine (LdT), tenofovir alafenamide fumarate (TAF), and tenofovir disoproxil fumarate (TDF) for preventing hepatitis B transmission in immune‐tolerant pregnant women with HBV infection.

**Methods:**

We conducted a retrospective cohort study involving women who had hepatitis B virus deoxyribonucleic acid (HBV DNA) ≥ 2 × 10^5 ^IU/mL and initiated LdT, TDF, or TAF to prevent mother‐to‐child transmission (MTCT). The primary endpoint was the safety of mothers and infants. The secondary endpoints were maternal HBV DNA reduction at delivery and MTCT rate.

**Results:**

A total of 96 patients were enrolled in the study (LdT group, *n *= 36; TDF group, *n* = 35; TAF group, *n *= 25). All infants received hepatitis B virus immunoprophylaxis. The MTCT rate was 0%([0 of 25] vs. [0 of 35] vs. [0 of 36], *p *> .05). No severe liver function damage occurred in any of the mothers. Babies delivered in all groups had prenatal ultrasound screening abnormalities, but abnormality rates were not statistically significant between groups.

**Conclusion:**

The application of TDF, TAF, or LdT to immune‐tolerant HBV‐infected pregnant women in middle–late pregnancy can successfully interrupt MTCT of the HBV virus. However, for all three groups of pregnant women who delivered babies with abnormal prenatal ultrasound screening, an expanded sample size may be needed for further observation.

**Abbreviations**
ALB: albumin
ALT: alanine aminotransferase
CK: creatine kinase
HBV DNA: hepatitis B virus deoxyribonucleic acid
HGB: hemoglobin
LdT: telbivudin
TAF: tenofovir alafenamide fumarate
TBA: total biliary acid
TBIL: total bilirubin
TDF: tenofovir disoproxil fumarate

## 1 INTRODUCTION

In 2016, the World Health Organization (WHO) counted that there are about 87 million chronic hepatitis B (CHB) patients in China, accounting for 1/3 of the world. Approximately 650,000 people died due to hepatitis B virus (HBV) related complications, which mostly included end‐stage liver diseases such as cirrhosis and hepatocellular carcinoma.^1^ HBV infection in China is mainly a perinatal or infantile infection, and infection in children under 6 years of age is more likely to become chronic.^2^ Therefore, prevention of mother‐to‐child transmission (MTCT) of HBV is an important measure to achieve the WHO target. Infections in children under 5 years of age have declined from about 5% between the 1980s and the early 21st century, before the introduction of vaccines, to just under 1% in 2019.^2^ However, even with combined hepatitis B vaccine and hepatitis B immunoglobulin (HBIG) immunization, infants born from pregnant women with high viral loads (>1 × 10^7 ^IU/mL) still have a prophylactic failure rate of 10%–30%.^3^ Therefore, targeted and effective interventions for pregnant women with high viral load, based on the current co‐immunization strategy, may be an effective way to further reduce MTCT of HBV. Since 2006, telbivudine (LdT), tenofovir disoproxil fumarate (TDF), and tenofovir alafenamide fumarate (TAF) in the United States have been listed by Food And Drug Administration as pregnancy class B drugs. But there are still few data comparing the efficacy and safety of these three drugs in maternal and infant blockade in immune‐tolerant pregnant women. This study aims to compare the efficacy and safety of TDF, TAF, and LdT in prevent MTCT of HBV, to improve the rational use of clinical drugs.

## 2 PATIENTS AND METHODS

### 2.1 General information

Pregnant women with CHB who attended the outpatient clinic of the Department of Infection of the Third Affiliated Hospital of Sun Yat‐sen University from January 2018 to October 2021, took antiviral drugs as prescribed and given births in our hospital. Inclusion criteria: (1) Diagnosed with CHB virus infection (positive for the hepatitis B surface antigen [HBsAg], positive for the hepatitis B e antigen, and serum HBV deoxyribonucleic acid [HBV DNA] ≥ 2 × 10^5 ^IU/mL). (2) Alanine aminotransferase (ALT) is normal. (3) Infants were immunized within 24 h after delivery with 100 IU of immunoglobulin and 10 ug of hepatitis B vaccine, and received hepatitis B vaccine at 1 and 6 months of age, respectively. (4) Signed an informed consent. (5) Completeness of clinical data ≥80%. Exclusion criteria: (1) Active hepatitis or co‐infection with other hepatitis virus or human immunodeficidency virus. (2) Previous pregnancy with a history of miscarriage, congenital neonatal malformations, or fetal malformations on ultrasound. (3) Previous history of antiviral therapy. (4) Previous renal dysfunction, cirrhosis, or liver decompensation, elevated serum ALT. (5) Have used nephrotoxic drugs, steroids, cytotoxic drugs, nonsteroidal anti‐inflammatory drugs, or immunomodulatory. (6) Poor treatment compliance.

### 2.2 Treatment method

A total of 96 pregnant women who were immune tolerant to HBV infection and started taking antihepatitis B drugs at 20–32 weeks of gestation were divided into three groups according to their option: LdT, TAF, and TDF. TDF treatment group 300 mg/day, TAF treatment group 25 mg/day, LdT treatment group 600 mg/day. Venous blood was collected before, during delivery, and 1 and 2 months after treatment, and HBV DNA and liver function (including ALT, aspartate aminotransferase[AST], albumin, total bilirubin [TBIL]) were measured, respectively. In addition, patients are routinely examined to record adverse reactions. The primary endpoint was the safety of mothers and infants. The secondary endpoints were maternal HBV DNA reduction at delivery and MTCT rate.

This research has been approved by the Ethics Committee of the Third Affiliated Hospital, Sun Yat‐sen University (approval number: II2023‐039). All patients gave their written informed consent before enrollment. This study was carried out according to the Declaration of Helsinki.

### 2.3 Statistical processing

SPSS statistical analysis software was used; Continuous variables were expressed as mean ± standard deviation; categorical variables were expressed as quantities and percentages. The units of HBV DNA quantification (IU/mL) were logarithmically transformed (log IU/mL). Measurements that conformed to normal distribution were expressed as mean ± standard deviation (x ± s), and comparisons between groups were made by *t* test or *χ* ^2 ^test, and comparisons before and after treatment in the same group were made by paired‐samples *t* test; comparisons between groups were made by one‐way analysis of variance, and comparisons between groups were made by Student‐Newman‐Keuls *t* test. Measurement data not conforming to normal distribution were expressed using M (P25, P75), and nonparametric tests were used. Count data were expressed as rate (%), and comparison of rates was performed using the *χ* ^2^ test; Fisher's exact test was used when the conditions of the *χ* ^2^ test were not met. *p* < .05 was used to indicate that the difference was statistically significant.

## 3 RESULTS

### 3.1 Clinical data from pregnant women before and 1 and 2 months after taking the medication

Based on the inclusion and exclusion criteria, we included 123 patients, 27 were lost in the follow‐up phase due to various reasons such as COVID‐19. All included patients met HBsAg positivity with HBV DNA ≥ 2 × 10^5 ^IU/mL, and there were no statistical differences in age, week of gestational on medication, ALT and Log_10_ HBV DNA among the three groups (*p* > .05) (Table 1). Based on the FIB‐4 scores (LdT group, 0.19 [0.15, 0.24]; TDF group, 0.17 [0.14, 0.22]; TAF group, 0.16 [0.13, 0.22], *p* > .05), we considered all patients to be in the noncirrhotic stage of the liver.

### 3.2 Changes in serum HBV DNA and ALT levels in three groups

There were no significant differences in ALT, TBIL, and reduction in HBV DNA in the three groups before treatment or at the time of delivery (*p *> .05) (Table 2), but the viral load was significantly reduced in the three groups (*p *< .05) (Table 3). Some mothers in each group showed HBV DNA negativity during delivery (Table 4).

### 3.3 Comparison of serum HBV marker changes in three groups of neonates

At birth, some infants in all three groups test positive for serum HBsAg but negative for HBV DNA. At 7 months of age, serum HBsAg was negative in all three groups (*p *> .05) (Table 5).

### 3.4 Safety of antiviral therapy during pregnancy and neonatal malformations in three groups

Among the three groups, only those in the TAF group had transient elevation of creatinine before delivery, and creatinine returned to normal after postpartum review.

All three groups of newborns had abnormal prenatal ultrasound screening, but there was no statistically significant difference (Table 6).

## 4 DISCUSSION

Previous studies have shown that in pregnant women with chronic HBV infection, the risk of passive‐active immunoprophylaxis failure begins to increase when the viral load ≥2 × 10^5 ^IU/mL.^4^ And with the increase of maternal HBV viral load, the infant HBV infection rate gradually increased. When the maternal viral load was 1 × 10^5‐^ ^7^ IU/mL, the predicted HBV infection rates were 6.6%, 14.6%, and 27.7%, respectively.^8^ The HBIG and hepatitis B vaccine program implemented in China can effectively reduce the vertical transmission of HBV from mother to child, but the prevention failure rate of infants born to pregnant women with high viral load (>1 × 10^7 ^IU/mL) is still 10%–30%.^3^

In general, mother–child transmission occurs mainly through three routes: intrauterine, during childbirth, and postpartum close contact with the mother. The most common routes are transmission in utero and during delivery, which explains the high rate of immunization failure in newborns after birth.^9^ Detailed routine antenatal testing reduces the risk of persistent HBV infection in the newborn.^5^ In pregnant women with positive HBV, maternal antiviral therapy during pregnancy and combined immunization of the newborn are the mainstay to preventing mother–child transmission.^9^ Low incidence of mother‐to‐child virus transmission. Antiviral drugs to interrupt the transmission of HBV is one of the key measures to prevent and control hepatitis B at the source, and it is of great significance to actively carry out researches on interruption of MTCT of HBV from mother to child.^6^

In this study, the use of TAF, TDF, or LdT for mother‐infant blockade therapy in the second and third trimesters of pregnancy significantly reduced HBV DNA load in the prenatal period, based on the principle of voluntary drug choice by pregnant women. There was no significant difference in HBsAg positivity between the three groups at 24 hours and 7 months after birth (*p *> .05). Pregnant women taking these three different antiviral drugs all had abnormal prenatal ultrasound screening rates, but there was no significant difference in the rate of malformations, but it should be noted that in newborns delivered to pregnant women taking TAF, prenatal ultrasound showed cardiac structural abnormalities and only one newborn had a cardiac ultrasound review during delivery, indicating that the patient had an atrial septal defect (4 mm).

Several studies have shown that many antiviral agents can further interrupt vertical transmission of HBV.^7^, ^10^, ^11^, ^12^ The 2019 guidelines for the prevention and treatment of CHB of China clearly states that TDF or LDT is recommended for pregnant women with CHB in the second or third trimester of pregnancy to further reduce MTCT of HBV.^13^ In 2021, a small controlled study reported that TAF and TDF effectively block the MTCT of hepatitis B. TAF was superior to TDF with regard to renal safety and breastfeeding.^14^ In 2020, other scholars reported that newborns born to pregnant women who took TDF and did not take antiviral drugs in the second and third trimesters of pregnancy for up to 6–7 years found that prenatal exposure to TDF did not affect long‐term growth and development, renal function, and bone development.^7^ In 2021, another follow‐up of newborns delivered to pregnant women taking LDT in the second and third trimesters of pregnancy for up to 3 years found that prenatal exposure to LdT can affect neurodevelopment.^15^ Few studies on the growth and development of newborns delivered to pregnant women taking antiviral drugs before delivery. This study found that newborns born to pregnant women taking three groups of drugs had abnormal prenatal ultrasound screening, and the highest proportion even reached 13.9%.

Some of the three groups of pregnant women taking antiviral drugs included in this study had abnormal prenatal ultrasound screening, but there was no significant difference in their malformation rates. However, it is worth noting that four of the neonates delivered by pregnant women taking TAF had structural cardiac abnormalities suggested by prenatal ultrasound screening, and one had a repeat fetal cardiac ultrasound at an outside hospital that did not show any significant abnormality. One additional neonate had a repeat cardiac ultrasound at delivery and at 6 months of age, suggesting that the patient had an atrial septal defect (4 mm). The remaining infants with abnormal ultrasound screening did not have their ultrasound reviewed. Upon consultation with the obstetrics and ultrasound departments, regular follow‐up was recommended for these infants and children, and the clinical significance of the abnormalities seen in these cases is unclear, so we will not be described in detail here.

This study is a systematic comparison of LdT, TDF, and TAF, and previous studies have been conducted to evaluate the efficacy and safety of NAs alone or both NAs in mid‐ and late pregnancy. Previous studies have evaluated the efficacy and safety of NAs alone or two NAs in late pregnancy. During the course of this study, all patients enrolled in the study were followed up by telephone. All patients enrolled in this study were followed up by telephone to verify that they had no congenital neonatal anomalies or ultrasound‐suggested fetal anomalies in previous pregnancies. For pregnant women with abnormalities on prenatal ultrasound screening, the follow‐up staff would focus on asking the mothers whether amniocentesis had been performed to assist in the diagnosis and treatment. Although the prenatal ultrasound screening was abnormal, some mothers did not undergo amniocentesis because they were concerned that the invasive procedure might increase the chance of MTCT of HBV. The most important finding of this study is that LdT, TDF, and TAF are effective in interrupting MTCT of HBV. The safety profile of the three drugs is almost the same.

This study has some limitations. First, as a retrospective study, the completeness of clinical data is poor. The results of the tests and examinations that were available to the eligible patients we included were more than 80%. Second, this study was a single‐center study with a small number of cases included, and the data collected may have been biased, affecting the accuracy of the results. Third, the follow‐up period for mothers and infants was short, and further observation is needed to see whether the mothers would cause liver function abnormalities after stopping antiviral therapy, abnormalities seen on prenatal ultrasound screening, and insufficient data on long‐term follow‐up in terms of infants. The effect of antiviral therapy plus combined immunoprophylaxis during pregnancy on the blockade of HBV MTCT also needs to be compared with combined immunoprophylaxis alone, and all these need to be further enlarged and studied in depth.

**Table 1:** Baseline clinical data from three groups of pregnant women.

	LdT	TDF	TAF
Patients	36	35	25
Age, years	29.41 ± 3.72	29.64 ± 3.22	30.38 ± 3.96
Gestational weeks	24.27 ± 3.63	24.40 ± 3.63	24.16 ± 5.23
ALT, U/L	20.50 (17.50, 37.75)	25.50 (15.25, 33.25)	18.00 (16.50, 32.00)
ALB, g/L	37.98 ± 2.15	38.23 ± 3.45	37.72 ± 2.80
TBIL, umol/L	5.10 (2.98, 7.04)	7.05 (5.17, 7.81)	4.10 (3.15, 4.55)
CK, U/L	58.50 (37.75, 88.00)	40.00 (26.00, 125.00)	44.00 (28.50, 56.00)
TBA, umol/L	2.40 (1.58,4.63)	2.65 (1.68, 6.08)	2.70 (1.45, 9.65)
Cr, mol/L	44.92 ± 7.01	46.47 ± 8.67	48.23 ± 6.94
HGB, g/L	114.84 ± 9.78	116.94 ± 14.60	113.72 ± 15.14
Log_10_ HBV DNA	7.53 ± 0.83	7.61 ± 0.82	7.84 ± 0.69

*Note*: AST is not included due to lack of enough data.

Abbreviations: ALB, albumin; ALT, alanine aminotransferase; CK, Cr, creatine kinase; TBA, total biliary acid; HGB, hemoglobin; HBV DNA, hepatitis B virus deoxyribonucleic acid; LdT, telbivudine; TAF, tenofovir alafenamide fumarate; TDF, tenofovir disoproxil fumarate.

**Table 2:** Information on pregnant women at the time of delivery.

	LdT	TDF	TAF	*p* Value
ALT, U/L	20.44 ± 8.85	20.65 ± 7.07	19.28 ± 8.66	>.05
ALB, g/L	36.32 ± 2.78	34.97 ± 2.35	35.15 ± 2.51	>.05
TBIL, umol/L	11.08 (8.84, 66.69)	3.60 (3.17, 4.85)	5.42 (4.38, 6.14)	>.05
CK, U/L	66.00 (49.50, 71.00)	67.00 (66.00, 71.00)	46.00 (31.00, 101.00)	>.05
TBA, umol/L	5.90 (3.80, 8.15)	6.50 (3.40, 7.60)	3.60 (2.85, 6.45)	>.05
ALP, U/L	165.00 ± 48.47	175.48 ± 55.71	166.25 ± 37.75	>.05
Cr, umol/L	51.07 ± 15.42	52.57 ± 10.39	47.88 ± 8.68	>.05
HGB, g/L	123.97 ± 13.38	118.29 ± 12.50	119.04 ± 13.26	>.05
Log_10 _HBV DNA	3.27 (1.63, 3.94)	2.44 (2.00, 3.38)	3.46 (2.92, 4.03)	>.05

Abbreviations: ALB, albumin; ALP, alkaline phosphatase; ALT, alanine aminotransferase; CK, Cr, creatine kinase; TBA, total biliary acid; TBIL, total bilirubin; HBV DNA, hepatitis B virus deoxyribonucleic acid; HGB, hemoglobin; LdT, telbivudine; TAF, tenofovir alafenamide fumarate; TDF, tenofovir disoproxil fumarate.

**Table 3:** Comparison of serum log_10_ HBV DNA before treatment and intrapartum (copies/mL).

Group	Patients	Before treatment	Delivery	*T* test	
				*T* value	*p* Value
LdT	36	7.53 ± 0.83	3.27 (1.63, 3.94)	18.89	<.05
TDF	35	7.61 ± 0.82	2.44 (2.00, 3.38)	11.73	<.05
TAF	25	7.84 ± 0.69	3.46 (2.92, 4.03)	20.49	<.05

Abbreviations: HBV DNA, hepatitis B virus deoxyribonucleic acid; LdT, telbivudine; TAF, tenofovir alafenamide fumarate; TDF, tenofovir disoproxil fumarate.

**Table 4:** Comparison of HBV DNA conversion rates in three groups of pregnant women during childbirth.

Group	Patients	HBV DNA loss
LdT	36	8 (22.2%)
TDF	35	3 (8.6%)
TAF	25	2 (8.0%)
*p* Value		>.05

Abbreviations: HBV DNA, hepatitis B virus deoxyribonucleic acid; LdT, telbivudine; TAF, tenofovir alafenamide fumarate; TDF, tenofovir disoproxil fumarate.

**Table 5:** Comparison of HBsAg positivity rates in three groups.

Group	Patients	24 h of birth	7 months
LdT	36	1 (2.8%)	0
TDF	35	2 (5.7%)	0
TAF	25	1 (4.0%)	0

Abbreviations: HBsAg, hepatitis B surface antigen; LdT, telbivudine; TAF, tenofovir alafenamide fumarate; TDF, tenofovir disoproxil fumarate.

**Table 6:** Comparison of abnormal rates of neonatal ultrasound screening in three groups.

Group	Patients	Deformity
LdT	36	5 (Heart, kidney, cranial suture, intestinal canal, nasal bone) (13.9%)
TDF	35	2 (Heart, ventricle, and 1 case of slow development) (5.9%)
TAF	25	3 (Heart) (12%)
*p* Value		.583

Abbreviations: LdT, telbivudine; TAF, tenofovir alafenamide fumarate; TDF, tenofovir disoproxil fumarate.

## AUTHOR CONTRIBUTIONS

Shufang Pan and Yingfu Zeng performed the research, analyzed the data, and wrote the paper. Chaoshuang Lin and Ying Zhang designed the research study and contributed essential reagents or tools. All authors have read and agreed to the published version of the manuscript.
